# Heat stress-induced gut dysbiosis and multi-organ injury: mechanisms and therapeutic modulation

**DOI:** 10.3389/fcimb.2026.1930660

**Published:** 2026-09-09

**Authors:** Zhuo Zhang, Jirui Wen, Ping Zhang, Yuhao Zou, Jiang Wu, Rong Yao

**Affiliations:** 1 Department of Emergency Medicine, West China Hospital, Sichuan University, Chengdu, China; 2 Department of Otolaryngology Head & Neck Surgery/Deep Underground Space Medical Center, West China Hospital, Sichuan University, Chengdu, China; 3 West China School of Basic Medical Sciences & Forensic Medicine, Sichuan University, Chengdu, China

**Keywords:** gut microbiota dysbiosis, heat stress, microbial metabolites, multi-organ injury, targeted intervention

## Abstract

Heat stress (HS) is an increasingly prevalent environmental and exertional challenge. In severe cases, HS may progress to heatstroke, a life-threatening clinical syndrome characterized by severe hyperthermia, systemic inflammation, and multi-organ dysfunction. This review synthesizes evidence from laboratory rodent models and suggests that gut microbiota dysbiosis may act as a mediator and amplifier of HS-induced pathology. Across diverse rodent models, HS remodels the gut microbiota by reducing microbial diversity. These compositional changes are accompanied by decreased short-chain fatty acids, altered bile acid profiles, and increased lipopolysaccharide burden, although the specific metabolites affected vary across models. Causal evidence from fecal microbiota transplantation and gnotobiotic experiments supports microbiota-dependent amplification of intestinal barrier failure, hepatic inflammation, and neuroinflammation. In contrast, evidence for the gut-reproductive, gut-kidney, gut-heart, gut-muscle, and gut-adipose axes remains predominantly associative or derived from interventional correlations without formal causality testing. Targeting the gut-organ axis through probiotics, prebiotics, antioxidants, or functional amino acids offers promising but largely preclinical adjunctive strategies, with rapid cooling and supportive care remaining the foundation of heatstroke management. Future research should prioritize temporally resolved human studies, multi-omics integration, and causal validation to define the translational potential of microbiome-directed interventions.

## 1 Introduction: Heat stress as a trigger of gut microbiota dysbiosis

Heat Stress (HS) refers to the physiological and pathological responses induced by exposure to high environmental or exertional heat, including metabolic disturbance, behavioral adaptation, intestinal barrier dysfunction, and organ injury (Gao et al., 2024; Cheng et al., 2026; Tang et al., 2023). The gut microbiota (GM) colonizing the gastrointestinal tract exerts multiple systemic functions in the host, including digestive, metabolic, immunological, homeostatic, and structural roles (Kluger et al., 1990; Fuller and Mitchell, 1999; Tappenden and Deutsch, 2007; Brown et al., 2012; Rosenberg and Zilber-Rosenberg, 2016; Liang et al., 2024). GM dysbiosis refers to a disruption of microbial community structure and function. Common features include reduced diversity, expansion of pathobionts, altered microbial metabolites, impaired mucosal interactions, and increased inflammatory potential (Shen et al., 2025). HS can disrupt this balanced ecosystem by reducing microbial diversity and promoting remodeling of the GM community.

Heat exposure alters the GM across mammalian models, while the affected taxa vary with host and protocol. In a pig model, chronic heat exposure at 33 °C for 21 days increased intestinal permeability, enriched taxa with pathogenic potential including *Asteroleplasma*, *Shuttleworthia*, and *Mycoplasma*, and reduced immune-associated *Coprococcus* and *Aeriscardovia* (Tang et al., 2022). Similarly, heat exposure at 35-38 °C for 7 days in rats persistently suppressed overall microbial diversity and markedly reshaped the community structure, with depletion of *Lactobacillus* and *Bacteroides* and enrichment of *Oscillospira* and *Clostridium* (Qu et al., 2021). Likewise, mice exposed to 40 °C for 2 hours per day over 7 days displayed a significant shift in β-diversity, characterized by reductions in *Candidatus Arthromitus* and *Ligilactobacillus murinus* and an increase in Lachnospiraceae bacteria (Wen et al., 2021a). Collectively, these studies suggest that HS is associated with a recurrent dysbiotic pattern characterized by increased pathobiont potential, higher endotoxin burden, and reduced abundance of taxa associated with host homeostasis (Xia et al., 2022).

Notably, HS exerts a dual impact on heat-tolerant opportunistic bacteria. It provides them with a competitive growth advantage and simultaneously enhances their virulence. Many opportunistic pathogens, including *Escherichia coli* and other Enterobacteriaceae, are inherently thermotolerant (Shahul Hameed et al., 2019). This thermotolerance may provide a competitive advantage under elevated temperatures. Pathogens including *Salmonella*, *Yersinia*, *Pseudomonas*, and pathogenic *E. coli* perceive host fever-range temperatures as an environmental cue (Brewer et al., 2021). This perception triggers upregulation of their virulence genes. Such virulence upregulation is notably more pronounced at 42 °C than at temperatures below 37 °C (Almblad et al., 2021). Thus, HS may alter microbial community structure and enhance the virulence potential of selected taxa, which may increase host susceptibility to systemic inflammatory responses.

## 2 Search strategy and evidence classification

The search strategy was developed with reference to Peer Review of Electronic Search Strategies (PRESS) guidance to improve transparency and reproducibility (McGowan et al., 2016). Four major databases were searched from inception to June 2026, including PubMed, Embase, Web of Science, and Scopus. The detailed search strategies with copies of system-generated search strings and the search results for individual databases are provided in **Supplementary Material S1**. This article was designed as a structured narrative review rather than a systematic review or meta-analysis. This decision reflects heterogeneity in heat exposure models, species, microbiota methods, and organ outcomes. Studies were prioritized when they assessed heat exposure together with GM or microbial metabolites and included organ injury outcomes or intestinal barrier outcomes. Studies were excluded when they did not address heat exposure, did not evaluate the GM or microbial metabolites, or lacked organ, inflammatory, metabolic, or barrier-related outcomes.

The key evidence across organ axes, including species, exposure protocols, microbiota changes, metabolites, target organs, and interventions, is summarized in **Table 1**. In this review, HS is used as an umbrella term for heat exposure that induces physiological stress and tissue injury. Heatstroke refers to the most severe clinical form of heat illness, characterized by extreme hyperthermia, neurological dysfunction, systemic inflammation, and multi-organ dysfunction (Zhang et al., 2024). Acute HS refers to short-term heat exposure without a fatal endpoint, whereas chronic HS refers to repeated or prolonged exposure of more than 7 days (Collier et al., 2017; Collier et al., 2019). Evidence from these models is discussed separately whenever possible because their pathophysiological relevance to clinical heatstroke differs.

**Table 1:** Summary of microbial and metabolic changes and organ outcomes across laboratory rodent heat stress models.

Study design	Species	Model category	Model protocol	Target organs	Intervention	Microbiota changes	Metabolite alterations	Reference
Interventional	mouse (C57BL/6J)	Heatstroke	40 °C, 50% RH to Heatstroke onset	Liver, Kidney	Heat acclimation	*Lactobacillus ↑;* *Helicobacter↓*	Deoxycholic acid↓	(Huang et al., 2023)
Interventional	mouse (ICR)	Acute heat stress	35 °C, 70% RH, 6d	Liver	Baicalin	*Lactobacillus ↑;* *Turicibacter ↓;* *Akkermansia ↓*	Lipopolysaccharide↓ (inferred from Lipopolysaccharide-binding protein ↓)	(Li et al., 2024)
Interventional	mouse (C57BL/6J)	Acute heat stress	40 °C, 65% RH, 2h/d × 7d	Liver	*Lactiplantibacillus plantarum* L19	*Lactobacillus ↑;* *Dorea ↑;* *Akkermansia ↓;* *Haemophilus ↓*	NA	(Wang et al., 2026)
Interventional	mouse (C57BL/6J)	Heatstroke	37.5 °C, 30% RH, forced running to collapse	Liver	Vancomycin	Clostridiales↓; Bacteroidales ↓	NA	(Oki et al., 2023)
Interventional	mouse (KM)	Acute heat stress	39 °C, 30% RH, 10h/d × 7d	Liver	Alhagi-honey	*Lachnoclostridium ↑*	NA	(Xu et al., 2024)
Interventional	mouse (BALB/c)	Acute heat stress	37 °C, 7d	Heart, Liver	*Ligilactobacillus salivarius*	*Lactobacillus ↑;* *Paenibacillus ↑;* *Alistipes ↓;* *Bacteroides ↓*	NA	(Yang et al., 2025)
Interventional	rat (SD)	Chronic heat stress	39 °C, 2.5h/d × 21d	Liver	Enzymatically treated mulberry leaf protein	*Faecalibaculum ↑;* *Romboutsia ↑*	NA	(Li et al., 2024)
Interventional	rat (SD)	Heatstroke	35 °C, 55% RH, treadmill to exhaustion	Liver	*Akkermansia muciniphila*	*Desulfovibrio ↑;* *Roseburia ↑*	Bile acids↑	(Yang et al., 2024)
Interventional	rat (SD)	Heatstroke	40 °C, 60% RH, treadmill to exhaustion	Lung, Liver, Kidney	Traditional Chinese Medicine (Huoxiang Zhengqi Dropping Pills)	*Lactococcus ↑;* *Streptococcus ↓*	NA	(Li et al., 2024)
Interventional	rat (SD)	Heatstroke	35 °C, treadmill to exhaustion	Liver	Short-chain fatty acids	*Bacteroides ↑;* *Rodentibacter ↑;* *Helicobacter ↑*	NA	(Yang et al., 2024)
Interventional	rat (SD)	Heatstroke	35 °C, 80% RH, treadmill to 42.5 °C	Liver, Kidney	*Bifidobacterium*	*Psychrobacter ↓*	NA	(Cai et al., 2026)
Interventional	mouse (C57BL/6)	Heatstroke	39.5 ± 0.5 °C, 65% RH, treadmill to exhaustion	Brain	Probiotic blend (*Lactobacillus* spp., *Bifidobacterium* spp.)	*Ligilactobacillus murinus ↑*	Pentanoic acid↑	(Xie et al., 2024)
Interventional	mouse (ICR)	Chronic heat stress	43 °C, 60% RH, 15min/d × 14d	Brain	β-Hydroxybutyric acid	*Lactobacillus ↑;* *Ruminococcus ↓;* *Prevotella ↓;* *Desulfovibrio ↓;* *Adlercreutzia ↓;* *Coprococcus ↓;* *Aerococcus ↑*	NA	(Sui et al., 2024)
Interventional	mouse (C57BL/6J)	Chronic heat stress	37 °C, 75–80% RH, 14d	Testis	*Lactiplantibacillus plantarum* 082	*Akkermansia ↑; Lactiplantibacillus ↑; Bifidobacterium ↑; Faecalibaculum ↑; Staphylococcus↓;* *Klebsiella ↓;* *Escherichia–Shigella group ↓; Mucispirillum ↓*	Acetic acid↑; Propionic acid↑; Butyric acid↑; Lipopolysaccharide↓	(Tu et al., 2026)
Interventional	mouse (ICR)	Acute heat stress	40 ± 1 °C, 40min/d × 5d	Testis	L-arginine	*Desulfovibrio ↑; Parabacteroides ↑*	NA	(Wang et al., 2025)
Interventional	mouse (ICR)	Acute heat stress	40 °C, 60% RH, 2h/d × 7d	Testis	*Enterococcus faecium* + *Clostridium butyricum*	*Staphylococcus ↓; Corynebacterium ↓; Mycoplasma ↓;* *Roseburia ↑;* *Blautia ↑*	Lipopolysaccharide↓	(Cai et al., 2023)
Interventional	mouse (ICR)	Chronic heat stress	38 °C, 2h/d × 16d (gestation)	Placenta, Fetus	Melatonin	*Alivibrio ↓;* *Butyricimonas ↑; Parasutterella ↑*	Lipopolysaccharide↓	(Wu et al., 2025)
Interventional	mouse (C57BL/6)	Chronic heat stress	38 °C, 2h/d × 14d	Adipose Tissue	*Lactiplantibacillus plantarum*	*Lactobacillus* ↑; *Desulfovibrio* ↓; *Akkermansia* ↓; *Ruminococcus* ↑; *Odoribacter* ↑	NA	(Deng et al., 2023)
Interventional	mouse (C57BL/6)	Chronic heat stress	37 °C, 14d	Skeletal Muscle	FMT (heat-exposed microbiota)	*Bacteroides ↑;* *Parabacteroides ↑;* *Bilophila ↑*	NA	(Guo et al., 2025)
Interventional	mouse (C57BL/6)	Chronic heat stress	40 °C, 2h/d × 14d	Skeletal Muscle	Sodium butyrate	*Parasutterella ↓;* *Dubosiella ↑;* *Allobaculum ↑;* *Ileibacterium ↑;* *Rikenella ↑;* *Lactobacillus ↑;* *Akkermansia ↑; Bifidobacterium ↑*	NA	(Lu et al., 2025)
Interventional	rat (Wistar)	Heatstroke	42 °C to Heatstroke onset	Lung, Liver, Kidney, Muscle	Sodium butyrate	*Ligilactobacillus murinus↑;* *Lactobacillus johnsonii ↓; Akkermansia muciniphila ↑*	NA	(Ke et al., 2025)
Interventional	rat (SD)	Acute heat stress	38 ± 1 °C, 75 ± 5% RH, 2h/d × 7d	Heart	Traditional Chinese Medicine (Sheng Mai San)	*Lactobacillus ↑;* *Globicatella ↓; Staphylococcus↓;* *Gemella ↓;* *Veillonella ↓*	NA	(Dong et al., 2025)
Observational	mouse (C57BL/6J)	Acute heat stress	35 °C, 70% RH, 6d	Liver	NA	*Escherichia–Shigella group↑;* *Acinetobacter ↑;* *Klebsiella ↑;* *Ruminiclostridium ↓;* *Blautia ↓*	Lipopolysaccharide↑	(He et al., 2021)
Observational	mouse (C57BL/6J)	Chronic heat stress	40 °C, 60% RH, 3h/d × 15d	Brain, Liver	NA	Firmicutes/Bacteroidetes ratio ↑	NA	(Roy et al., 2024)
Observational	mouse (C57BL/6J)	Chronic heat stress	38 °C, 2h/d × 14d	Testis	NA	*Asticacaculis ↑;* *Enhydrobacter ↑; Stenotrophomonas ↑; Enterococcus ↓; Pleomorphomonas ↓*	NA	(Cao et al., 2023)
Observational	rat (SD)	Acute heat stress	42 °C, 4h/d × 3d	Testis	NA	*Bifidobacterium ↑;* *Facklamia ↑*	NA	(Li et al., 2026)
Observational	Siberian hamster	Chronic heat stress	30 ± 1 °C, 10wk	Testis, Ovary, Uterus	NA	*Corynebacterium ↑; Lactobacillus ↑; LimosiLactobacillus ↑*	NA	(Shen et al., 2024)
Observational	mouse (ICR)	Chronic heat stress	38 °C, 2h/d × 15d (gestation)	Placenta, Fetus	NA	*Lactobacillus↓;* *AlloPrevotella↓;* *Mucispirillum↓;* *Rikenella↑;* *Alistipes↑;* Firmicutes/Bacteroidetes ratio ↑	Lipopolysaccharide↑; Short-chain fatty acids↓	(Zheng et al., 2023)

In observational studies, changes represent heat stress versus control. In interventional studies, changes represent intervention versus heat stress. Arrows indicate changes in relative abundance of microbial taxa or metabolite concentrations. Specifically, ↑ denotes an increase, whereas ↓ denotes a decrease. Taxonomic levels are reported as described in the original studies. Taxonomic alterations are reported at the level provided in the original studies, including phylum, order, family, genus, species, and unresolved 16S-based taxonomic groups. NA, not available. RH, relative humidity. FMT, fecal microbiota transplantation.

Model classification: Heatstroke models are defined by progression to core hyperthermia-induced collapse or loss of consciousness. Acute heat stress models are characterized by short-term daily exposure of 7 days or less without fatal endpoints. Chronic heat stress models involve repeated or prolonged exposure of more than 7 days.

The principal conclusions of this review are therefore derived from laboratory rodent evidence. Most mechanistic studies have used mice and rats, and these models provide the most complete evidence for causal relationships involving GM, microbial metabolites, and organ injury. When studies in mammalian livestock or avian species are cited for contextual support, the species and study setting are stated explicitly with specification of the heat exposure protocol or production environment. Such studies are treated only as supportive or hypothesis-generating evidence and are not included in **Tables 1**, **2**. **Table 2** summarizes the evidence hierarchy for GM involvement in HS-associated multi-organ injury. The evidence hierarchy distinguishes three levels, namely association, intervention, and mechanistic evidence. Each study is listed once and assigned to the highest evidence level it supports.

**Table 2:** Evidence hierarchy for gut microbiota involvement in heat stress–associated multi-organ injury in laboratory rodents.

Target organ	Species	Model category	Association (A)	Concordant intervention (I)	Microbiota-dependent evidence (M)
Liver	Mice	Heatstroke; Acute heat stress	NA	(Huang et al., 2023; Xu et al., 2024; Yang et al., 2025; Wang et al., 2026)	(Oki et al., 2023)
	Pregnant mice	Acute heat stress	(He et al., 2021)	(Li et al., 2024)	NA
	Aged mice	Chronic heat stress	(Roy et al., 2024)	NA	NA
	Rats	Heatstroke; chronic heat stress	NA	(Li et al., 2024; Li et al., 2024; Yang et al., 2024; Ke et al., 2025; Cai et al., 2026)	(Yang et al., 2024)
Kidney	Mice	Heatstroke	NA	(Huang et al., 2023)	NA
	Rats	Heatstroke	NA	(Li et al., 2024; Ke et al., 2025; Cai et al., 2026)	NA
Heart	Mice	Acute heat stress	NA	(Yang et al., 2025)	NA
	Rats	Acute heat stress	NA	(Dong et al., 2025)	NA
Brain	Mice	Heatstroke; chronic heat stress	NA	(Xie et al., 2024)	(Sui et al., 2024)
	Aged mice	Chronic heat stress	(Roy et al., 2024)	NA	NA
Testis	Mice	Acute and chronic heat stress	(Cao et al., 2023)	(Cai et al., 2023; Tu et al., 2026)	(Wang et al., 2025)
	Rats	Acute heat stress	(Li et al., 2026)	NA	NA
	Siberian hamsters	Chronic heat stress	(Shen et al., 2024)	NA	NA
Placenta and fetus	Pregnant mice	Chronic heat stress	(Zheng et al., 2023)	NA	(Wu et al., 2025)
Adipose tissue	Mice	Chronic heat stress	NA	(Deng et al., 2023)	NA
Skeletal muscle	Mice	Chronic heat stress	NA	(Lu et al., 2025)	(Guo et al., 2025)
	Rats	Heatstroke	NA	(Ke et al., 2025)	NA

Evidence was classified into three mutually exclusive levels according to the highest level achieved by each study. Association evidence (A) indicates observational relationships between heat-related alterations in microbial composition or metabolites and organ injury, without causal inference. Concordant intervention evidence (I) indicates that an intervention improved microbial or metabolite readouts together with organ phenotypes, but did not establish microbiota dependence or a direct mechanism. Mechanistic evidence (M) includes causal evidence obtained through fecal microbiota transplantation, microbiota depletion, germ-free or pseudo-germ-free models, defined microbial intervention, or direct validation of a specified microbial product, metabolite, receptor, or downstream pathway using rescue, blockade, or targeted manipulation. Each study was assigned only to the highest evidence level it achieved. NA, not available.

## 3 Intestinal barrier failure and GM dysbiosis: interacting pathways in multi-organ injury

The gastrointestinal tract has a large surface area in contact with the external environment, and its intestinal epithelium forms a crucial physical and biochemical barrier separating the luminal microbiota from the mucosal immune system (Furness et al., 1999; Bai et al., 2021). The pathophysiology of HS-induced intestinal injury involves multiple concurrent and interconnected pathways. Direct thermal cytotoxicity disrupts enterocyte membrane integrity and mitochondrial function and can trigger reactive oxygen species (ROS) production early during severe heat exposure (Tang et al., 2023; Sun et al., 2024). Simultaneously, splanchnic vasoconstriction can markedly reduce intestinal blood flow and can promote tissue hypoxia and ischemia-reperfusion injury early after HS exposure (Ogden et al., 2020a; Cinca-Morros and Álvarez-Herms, 2024). Intestinal hypoxia can also induce local inflammation through barrier-independent pathways (Lian et al., 2020). These thermal and ischemic insults compromise epithelial integrity and increase paracellular permeability within minutes to hours (Ogden et al., 2020a). Furthermore, HS activates coagulation cascades through both extrinsic and intrinsic pathways, leading to microvascular thrombosis and tissue necrosis that are independent of microbial translocation (Armstrong et al., 2018; Sun et al., 2024). As a result, increased permeability facilitates translocation of luminal bacterial products, particularly lipopolysaccharide (LPS), into the portal and systemic circulations. This translocation triggers local and systemic inflammatory responses that can further disrupt the microbial ecosystem over days to weeks, promoting outgrowth of pathobionts and depletion of taxa associated with homeostatic functions (Wen et al., 2021b; Ringseis and Eder, 2022). Fecal microbiota transplantation (FMT) from heat-stressed animals may transfer intestinal injury-related phenotypes to recipient animals. Hu et al. demonstrated that transplanting microbiota from heat-stressed pigs into pseudo-germ-free mice recapitulated signs of intestinal inflammation and damage, including lymphocyte infiltration and reduced colonic mucosal height (Hu et al., 2022). This finding suggests that established dysbiosis may contribute to persistence and amplification of intestinal injury (Ringseis and Eder, 2022).

**Figure 1:**
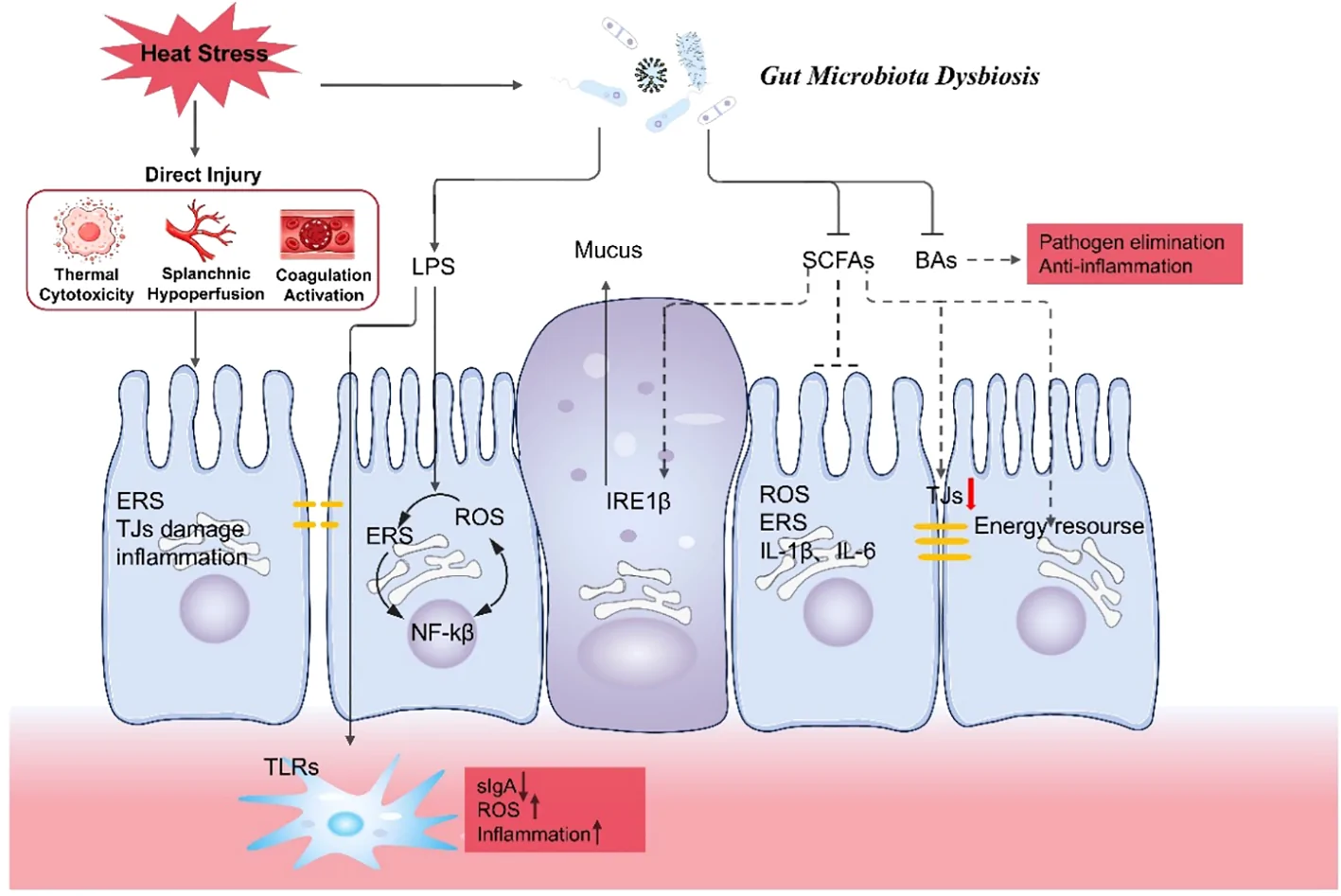
Proposed mechanisms of intestinal barrier disruption and gut microbiota-mediated amplification under heat stress. This schematic contrasts established direct heat-induced pathology with proposed microbiota-dependent modulatory pathways. The left panel shows direct epithelial damage from thermal cytotoxicity, splanchnic hypoperfusion, ischemia, and coagulation activation. The right panel depicts concurrent gut microbiota dysbiosis, characterized by reduced short-chain fatty acid-producing taxa and altered bile acid profiles. These shifts amplify barrier injury via energy depletion, impaired antioxidant and anti-inflammatory signaling, and reduced mucus production. Concurrently, pathobiont overgrowth increases local lipopolysaccharide burden. Increased permeability allows bacterial translocation, triggering Toll-like receptor-mediated inflammation and establishing a feedforward injury loop. ERS, endoplasmic reticulum stress; ROS, reactive oxygen species; LPS, lipopolysaccharide; TLRs, Toll-like receptors; SCFAs, short-chain fatty acids; BAs, bile acids. TJs, tight junctions; sIgA, secretory immunoglobulin A; NF-κB, nuclear factor kappa B; IRE1β, inositol-requiring enzyme 1 beta.

GM dysbiosis contributes to intestinal barrier failure through multiple mechanisms during HS (**Figure 1**). Commensals such as *Lactobacillus* and *Bifidobacterium* contribute to intestinal homeostasis by interacting with the mucus layer and intestinal epithelial cells (IEC), thereby supporting colonization resistance against pathogens (Dempsey and Corr, 2022; Gavzy et al., 2023). *Lactobacillus* species exert anti-inflammatory effects and promote IEC proliferation (Wu et al., 2020). The p40 protein secreted by *Lactobacillus* protects IECs through epidermal growth factor receptor activation (Shen et al., 2018). Concurrently, a reduction in microbial metabolites, which directly and indirectly modulate host physiology (Huang et al., 2021), contributes to barrier failure. HS reduces the abundance of short-chain fatty acids (SCFAs)-associated taxa, including *Lactobacillus*, *Bacteroides*, and *Clostridium butyricum*, thereby decreasing colonic levels of butyrate, acetate, and propionate (Koh et al., 2016; Xiong et al., 2020; Su et al., 2022). Importantly, butyrate has been tested as a direct intervention in a controlled rodent HS model. In male rats subjected to experimental heatstroke in a heating chamber, butyrate treatment reduced local and systemic inflammation, oxidative stress, and apoptosis while improving organ injury and survival (Ke et al., 2025). Butyrate is a major energy substrate for colonocytes, whereas acetate and propionate contribute to luminal acidification and systemic metabolism. Beyond these metabolic roles, depending on their concentration, receptor engagement, target tissue, and experimental model, individual SCFAs can regulate claudin-1 and zonula occludens-1 (ZO-1), stimulate mucin secretion, and support barrier integrity (Huang et al., 2021; Su et al., 2022). Butyrate oxidation in IECs increases epithelial oxygen consumption, stabilizes hypoxia-inducible factor, and promotes tight junction expression in experimental models of intestinal inflammation (Hirota et al., 2010; Makki et al., 2018; Pral et al., 2021).

Bile acids (BAs) metabolism represents a second metabolite pathway, distinct from SCFAs, through which heat-induced GM dysbiosis may influence intestinal barrier integrity. However, the direction and mechanism of BA effects depend on the specific BA species and the experimental context. In a heat acclimation model, four weeks of pre-exposure at 34 °C before HS reduced deoxycholic acid (DCA), increased tight junction protein expression, and attenuated intestinal injury (Huang et al., 2023). These findings suggest that reducing DCA may be protective. In contrast, chronic heat exposure produced the opposite pattern. Mice exposed to high temperature and humidity for 45 days showed increased serum lithocholic acid (LCA), decreased colonic claudin-1 and ZO-1 expression, and increased permeability. FMT from heat-exposed donors into germ-free recipients reproduced the higher serum LCA and barrier phenotype, indicating that GM changes drive this BA shift (Weng et al., 2024). In human colonic T84 cells, LCA and chenodeoxycholic acid (CDCA) exhibited opposite effects on epithelial integrity, with LCA restoring barrier function (Sarathy et al., 2017). DCA, in contrast, consistently impairs barrier function. Prolonged exposure to physiological concentrations of DCA decreased transepithelial electrical resistance and increased paracellular permeability in Caco-2 cell monolayers (He et al., 2024). DCA also downregulated the expression of multiple tight junction and adherens junction genes and increased intracellular ROS production (Zeng et al., 2022). Thus, although BA metabolism is perturbed during HS, the functional consequences for intestinal barrier integrity differ markedly across BA species and experimental models.

## 4 Multi-organ injury mediated by GM dysbiosis

The strength of evidence differs across organ axes. Interpretation should consider whether the data come from heatstroke models, acute or chronic HS models, direct microbiome manipulation, or extrapolation from non-heat-related microbiome literature. The gut-liver axis is prioritized because of the direct anatomical connection through the portal circulation and the relatively stronger experimental evidence linking microbial translocation, LPS signaling, certain SCFA deficiencies, BA dysregulation, and liver injury. The gut-brain axis is emphasized because neurological dysfunction is central to heatstroke diagnosis. Emerging studies also suggest links among blood-brain barrier (BBB) injury, neuroinflammation, microbial metabolites, and cognitive impairment. Evidence for the gut-reproductive, gut-kidney, gut-heart, gut-muscle, and gut-adipose axes is biologically plausible but less mature, and many mechanisms remain extrapolated from HS models or broader microbiome literature. Therefore, these sections should be interpreted as proposed mechanistic frameworks rather than established causal pathways.

### 4.1 The gut-liver axis

**Figure 2:**
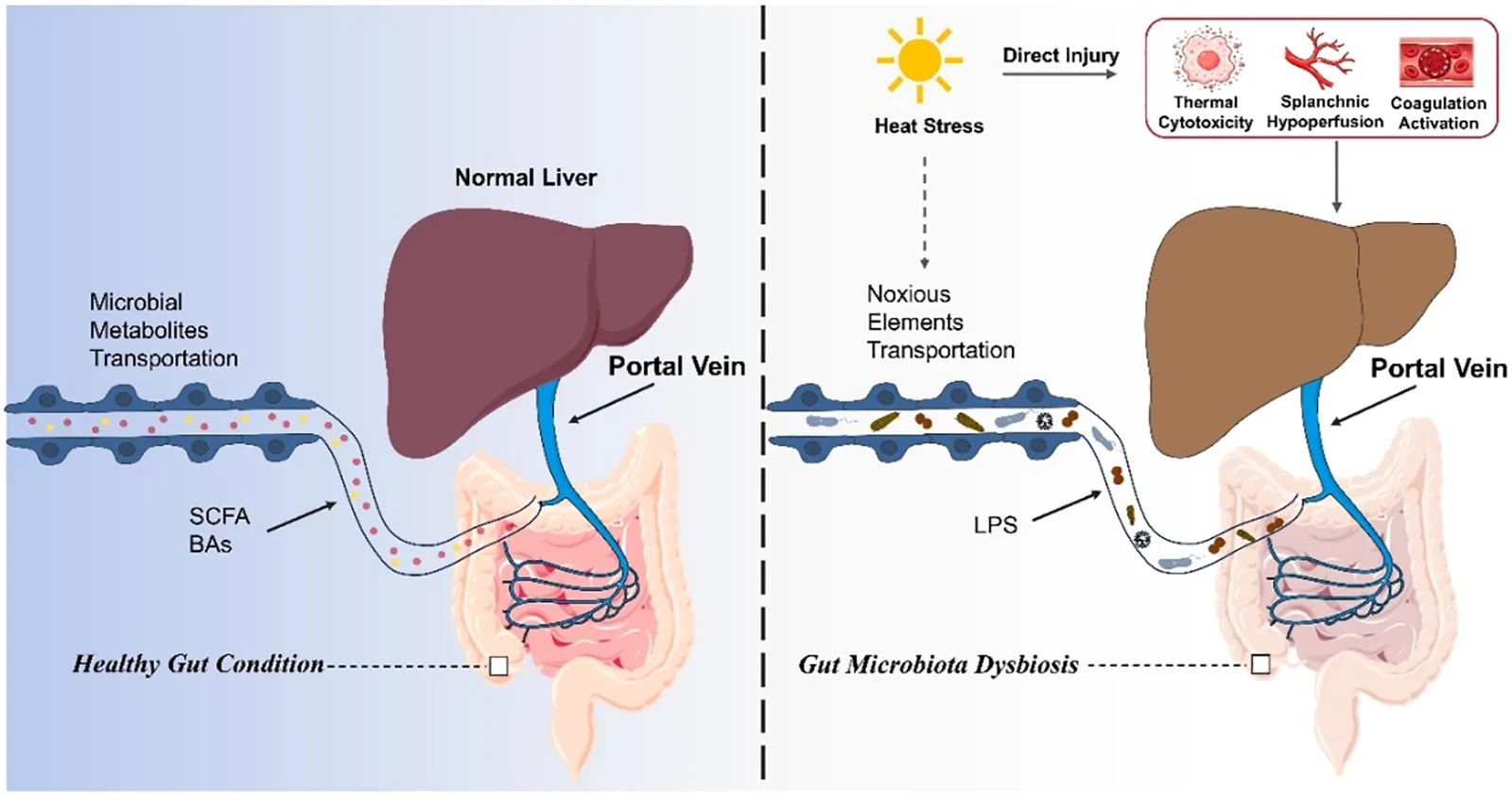
Proposed gut-liver axis in health and heat stress. The left panel shows a healthy state in which a balanced gut microbiome and intact intestinal barrier limit entry of harmful microbial products into the portal circulation. The right panel shows a proposed HS-associated pathway in which GM dysbiosis and barrier disruption permit microbial translocation to the liver through the portal vein and may promote inflammation and liver injury. LPS, lipopolysaccharide; SCFAs, short-chain fatty acids; BAs, bile acids.

The gut and liver interact via several pathways. One way is their anatomical association, where venous blood from the small and large intestine drains into the portal vein, which provides 75% of the liver’s blood supply. These organs also interact via the microbiota, as the liver is the first organ to encounter enterally absorbed nutrients and microbial metabolites (Hsu and Schnabl, 2023). The liver, in turn, regulates the gut by secreting bile containing BA, immunoglobulin A, and antimicrobial molecules directly into the small intestine, which completes the cycle by modulating the microbiota. Under physiological conditions, this gut–liver axis is important for liver homeostasis, whereas HS can disrupt this interaction. During HS, hyperthermia, hypoperfusion, and coagulation abnormalities directly injure the liver. Intestinal barrier disruption can subsequently increase the portal delivery of microbial products and metabolites, which modify hepatic immune and metabolic responses (**Figure 2**).

Evidence from experimental models supports the involvement of gut-derived signals in the modulation of HS-induced liver injury (Roy et al., 2024; Yang Z. et al., 2024). In a mouse heatstroke model, FMT from heatstroke donors into recipient mice aggravated subsequent liver injury, indicating that dysbiosis itself can amplify hepatic damage. The same study linked reduced *Ligilactobacillus murinus* abundance and lower xanthohumol levels to organ injury and showed that xanthohumol supplementation suppressed NLRP3 transcription through hnRNPK binding and attenuated macrophage pyroptosis (Huang et al., 2025). In late gestational mice exposed to 37 °C for 7 days, baicalin reduced hepatic injury alongside alterations in GM composition, intestinal barrier integrity, lipid metabolism, and inflammatory signaling (Li et al., 2024). These interventions provide concordant evidence across gut and liver endpoints.

#### 4.1.1 Inflammation and hepatocyte death

Direct thermal injury and hepatic hypoperfusion initiate oxidative stress and sterile inflammatory signaling during HS. A compromised intestinal barrier allows gut-derived pathogens and their products to enter the portal circulation. LPS binds to Toll-like receptor 4 (TLR4) on Kupffer cells, the main liver-resident macrophages, and triggers an nuclear factor kappa B (NF-κB) -mediated inflammatory cascade that culminates in hepatocyte apoptosis and necrosis (Heled et al., 2013). This LPS-driven inflammatory response is critically amplified by the concurrent loss of microbial-derived anti-inflammatory factors. HS-induced GM dysbiosis depletes SCFA-producing bacteria (Cui and Gu, 2015; Zhou et al., 2020; Shih et al., 2023). The resulting deficiency in butyrate and propionate removes their potent inhibitory effect on NF-κB activation, thereby deregulating inflammatory signaling in the liver (Hirota et al., 2010; He et al., 2019; Huang et al., 2020). In male rats exercised to exhaustion at 35 °C, pretreatment for 5 days with a mixture of sodium acetate, sodium propionate, and sodium butyrate attenuated histological liver and colon injury, reduced serum ALT and AST concentrations, and altered GM composition. In an FMT experiment, antibiotic-treated rats receiving feces from SCFA-pretreated donors developed milder liver and colon injury than recipients of feces from heat-exposed donors without SCFA pretreatment (Yang et al., 2024).

#### 4.1.2 Metabolic dysregulation and steatosis

Concurrently, GM dysbiosis may contribute to hepatic metabolic dysfunction by altering specific microbial metabolites during HS. In pregnant mice exposed to 37 °C during late gestation, HS coincided with lower serum insulin and triglyceride concentrations, higher nonesterified fatty acid and β-hydroxybutyrate concentrations, reduced hepatic mitochondrial gene expression, and a reduction in SCFA-producing bacteria (He et al., 2021). Male rats running to exhaustion at 35 °C developed hepatic steatosis, and SCFA pretreatment attenuated liver injury in this model (Yang et al., 2024). Propionate, a gluconeogenic precursor, has been shown to both promote and inhibit gluconeogenesis depending on the context and receptor pathway involved (Jiang et al., 2023).

BA metabolism provides a second route, in addition to SCFAs, through which intestinal microbial changes may influence hepatic metabolism. Gut microbial deconjugation and dehydroxylation alter BA composition and the availability of ligands for farnesoid X receptor (FXR) and G protein-coupled bile acid receptor 1 (TGR5), which regulate lipid and glucose metabolism, inflammation, and energy expenditure (Li and Chiang, 2014; Wahlström et al., 2016). In male mice exposed to 35 °C and 85% relative humidity for three days, hepatic BA profiling showed increased lithocholic acid and decreased glycodeoxycholic acid. Hepatic lithocholic acid correlated positively with fecal *Aliihoeflea, Azohydromonas, and Halomonas* (Wang et al., 2021). In male mice exposed to 33 °C and 95% relative humidity for 45 days, *Ligilactobacillus murinus* abundance decreased and serum lithocholic acid increased. FMT from exposed donors reproduced the lithocholic acid increase in germ-free recipients (Weng et al., 2024). Supportive evidence from poultry studies also supports this association. In Arbor Acres broilers maintained at 32 °C from 21 to 42 days of age, a mixed BA supplement reduced hepatic triglycerides and the expression of sterol regulatory element-binding protein 1c (SREBP-1c) and fatty acid synthase (FAS) (Yin et al., 2021). These findings support an association between BA alterations and hepatic lipid accumulation under HS. A pathway linking a defined bacterial taxon, a specific BA, receptor engagement, and hepatic injury has not yet been validated in a controlled rodent HS model.

### 4.2 The gut-brain axis

HS markedly affects brain structure and function, including neuronal loss, convulsions, neurogenic deficits, memory and cognitive dysfunction, and neuroinflammation (Lee et al., 2015; Cedeno Laurent et al., 2018). Recent findings support a model in which GM modulation and direct host effects jointly influence neurological outcomes after HS. For example, Xie et al. reported that exertional HS was associated with long-term cognitive impairment, GM alterations, and reduced SCFAs. Importantly, probiotic supplementation with *Lactobacillus* spp. can mitigate this impairment (Xie et al., 2024). Furthermore, Sui et al. demonstrated that FMT combined with β-hydroxybutyric acid partially reversed GM disruption in HS mice, suppressed microglial overactivation, and alleviated central nervous system (CNS) inflammation (Sui et al., 2024).

#### 4.2.1 Neuroinflammation and barrier disruption

Severe heat exposure can directly impair BBB integrity by downregulating tight junction proteins such as ZO-1, occludin, and claudin-5 in brain microvascular endothelial cells, with increased BBB permeability indicated by perivascular IgG extravasation and edema (Li et al., 2025). This barrier disruption facilitates the entry of circulating LPS and pro-inflammatory cytokines into the brain parenchyma, thereby triggering neuroinflammation (Zhou et al., 2026).

**Figure 3:**
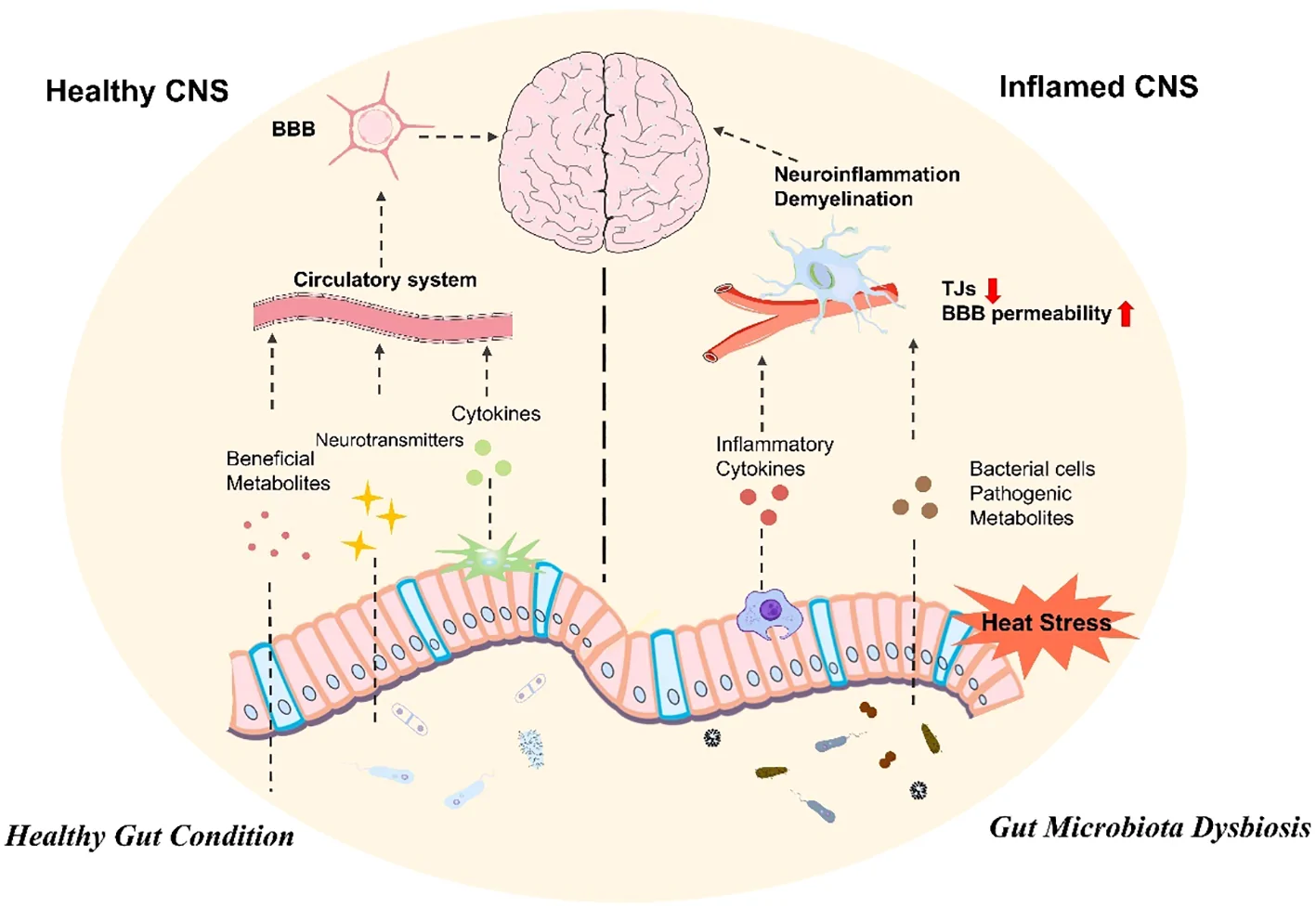
Gut-brain axis under health and heat stress. Left panel shows the healthy condition. A stable microbiota and intact BBB limit pathogen entry and neuroinflammation; beneficial metabolites and anti-inflammatory cytokines support CNS integrity. Right panel illustrates HS-induced BBB disruption. HS compromises BBB integrity by downregulating tight junction proteins. HS-induced GM dysbiosis and increased BBB permeability permit pathogenic metabolites and pro-inflammatory cytokines to enter cerebral circulation, provoking neuroinflammation, demyelination and neuronal injury. TJs, tight junctions; BBB, blood-brain barrier; CNS, central nervous system.

GM-related processes may amplify intestinal and BBB injury through increased permeability and systemic exposure to microbial products (**Figure 3**). In adult male mice, chronic mild HS at 33 °C for 2 h daily over 7 days induced intestinal hyperpermeability, elevated plasma LPS, BBB leakage, hippocampal inflammation, oxidative injury, and cognitive deficits (Lin et al., 2024). A complementary observation in 24-month-old male mice exposed to 40 °C for 3 h daily over 15 days showed that heat exposure increased the fecal Firmicutes-to-Bacteroidetes ratio and intestinal IL-1β. These changes coincided with elevated circulating IL-1β and IL-6, reduced cerebral claudin-5 and CD31 colocalization, and increased cerebral IL-6 (Roy et al., 2024). Together, these findings from distinct mouse models support an association between heat-induced gut dysbiosis, systemic inflammation, and BBB disruption. β-Hydroxybutyric acid (BHBA) provides intervention evidence for a microbiota-gut-brain axis pathway. In male ICR mice exposed to 43 °C for 15 min daily over 14 days, BHBA at 200 mg/kg per day reduced microglial activation and suppressed IL-1β, IL-6, NF-κB expression in the brain (Sui et al., 2024). FMT from BHBA-pretreated donors into heat-exposed recipients effectively reversed GM disruption and attenuated microglial hyperactivation.

#### 4.2.2 Neurotransmitter dysregulation and cognitive impairment

Heat exposure directly disturbs hypothalamic neurotransmission and hippocampal signaling. In male rats exposed to 45 °C, moderate HS (core temperature 40 °C) increased hypothalamic norepinephrine and reduced serotonin, whereas severe HS (core temperature 42 °C) increased hypothalamic epinephrine, dopamine, and glutamate and activated NF-κB, IL-1β, and COX-2 (Chauhan et al., 2017). These findings link direct heat-induced neuroinflammation to disruption of neurotransmitter systems involved in thermoregulation.

In addition to these direct effects, emerging evidence from rodent models supports an association between GM alterations and cognitive impairment following severe heat exposure. In an exertional heatstroke model, cognitive deficits persisted after intestinal and hippocampal injuries had largely resolved, coinciding with reduced Lactobacillaceae and altered fecal metabolites including increased pentanoic acid (Xie et al., 2024). A probiotic intervention in the same model improved cognitive performance and enhanced hippocampal brain-derived neurotrophic factor (BDNF) pathway molecule expression, supporting a microbiota-dependent mechanism (Xie et al., 2024). Additional evidence from a dose-dependent study in rats exposed to 25 °C, 30 °C, 35 °C, or 40 °C for four hours daily over 15 days showed that incremental heat exposure reduced fecal acetate, propionate, and butyrate and induced intestinal barrier disruption, hippocampal damage, and cognitive impairment (Zhuo et al., 2025).

HS-induced GM dysbiosis may influence hypothalamic–pituitary–adrenal (HPA) axis activity and circulating glucocorticoid levels (Wen et al., 2021b). Activation of this axis begins with corticotropin-releasing factor release from the hypothalamus, stimulating adrenocorticotropic hormone secretion from the anterior pituitary, which ultimately triggers corticosteroid release into circulation. These corticosteroids may subsequently affect the gastrointestinal tract by interacting with enteric cells, leading to cytokine release. The cytokines can then act on the brain to influence mood, appetite, cognition, and emotion (Cryan and Dinan, 2012). Notably, elevated glucocorticoids can in turn exert effects on GM composition and increase gastrointestinal permeability, creating a feedback loop that amplifies both neuroendocrine and microbial dysregulation (de Punder and Pruimboom, 2015; Wen et al., 2021b).

Beyond the HPA axis, SCFAs are positively correlated with choline levels in the anterior cingulate cortex, a region involved in cognitive processing (He et al., 2018). Individual SCFAs play distinct roles. For example, microbial-derived acetate can stimulate ghrelin secretion to promote cerebral gamma-aminobutyric acid (GABA) production, which is involved in emotion regulation and learning (Fragas et al., 2022; Facchin et al., 2024). Butyrate demonstrates antidepressant effects, influences social behavior, and has shown positive impacts on pathology and memory in preclinical Alzheimer’s disease models (Govindarajan et al., 2011). Therefore, the HS-induced deficiency in these SCFAs may constitute a mechanism linking gut dysbiosis to cognitive impairment.

A large proportion of peripheral serotonin is synthesized in the gut, and this process can be influenced by the GM (Bremshey et al., 2024; Sanidad et al., 2024). Specific bacteria, including *Escherichia coli*, *Lactobacillus*, and *Bifidobacterium*, may participate in metabolizing tryptophan (Trp) for serotonin production (Ala, 2022). Peripheral serotonin, which is influenced by gut microbial Trp metabolism, does not cross BBB. Rather, effects on central neurotransmission are likely mediated through vagal signaling, immune mediators, and Trp availability via the kynurenine pathway (Bremshey et al., 2024). During HS-induced systemic inflammation, Trp metabolism may shift toward the kynurenine pathway, producing neuroactive metabolites such as quinolinic acid and 3-hydroxykynurenine that can exacerbate excitotoxicity and oxidative stress (Wang et al., 2015). Additionally, the GM can metabolize Trp into indole derivatives that may exert anti-inflammatory and neuroprotective effects through aryl hydrocarbon receptor signaling (Iwaniak et al., 2024). In dysbiotic states, production of protective indole derivatives may decline, thereby weakening anti-inflammatory signaling in the intestine and CNS. This dual dysregulation, excessive kynurenine pathway activation and deficient indole-mediated protection, may contribute to the neurocognitive impairments observed during HS. Recent studies suggest a role for Trp in thermoregulation during HS, possibly through alleviating oxidative stress, and identify it as a determinant of mood and fear-related behaviors. Consequently, GM dysbiosis may contribute to neurochemical imbalance and cognitive or affective impairment during HS through proposed immune, endocrine, vagal, and metabolite-dependent pathways.

### 4.3 The gut-reproductive axis

HS exerts detrimental effects on the male reproductive system, including testicular damage, impaired spermatogenesis, and reduced sperm quality (Durairajanayagam et al., 2015). Pregnant individuals are also particularly vulnerable to HS, with adverse outcomes including preterm delivery, stillbirth, placental insufficiency, and abnormal fetal growth (Kuehn and McCormick, 2017; He et al., 2018; Chersich et al., 2020). The GM has emerged as a potential therapeutic target for mitigating heat-associated reproductive injury in preclinical models. Wang et al. demonstrated that L-arginine supplementation improved spermatogenesis in heat-stressed mice and that FMT from L-arginine-treated donors reproduced the protective effect, supporting a microbiota-dependent contribution (Wang et al., 2025). In addition, Wu et al. used pseudo-germ-free and FMT approaches to show that the protective effect of melatonin on fetal intrauterine growth under high temperature exposure was partly dependent on the GM (Wu et al., 2025).

#### 4.3.1 Gut-testis axis: inflammation, oxidative stress, and metabolic disruption

**Figure 4:**
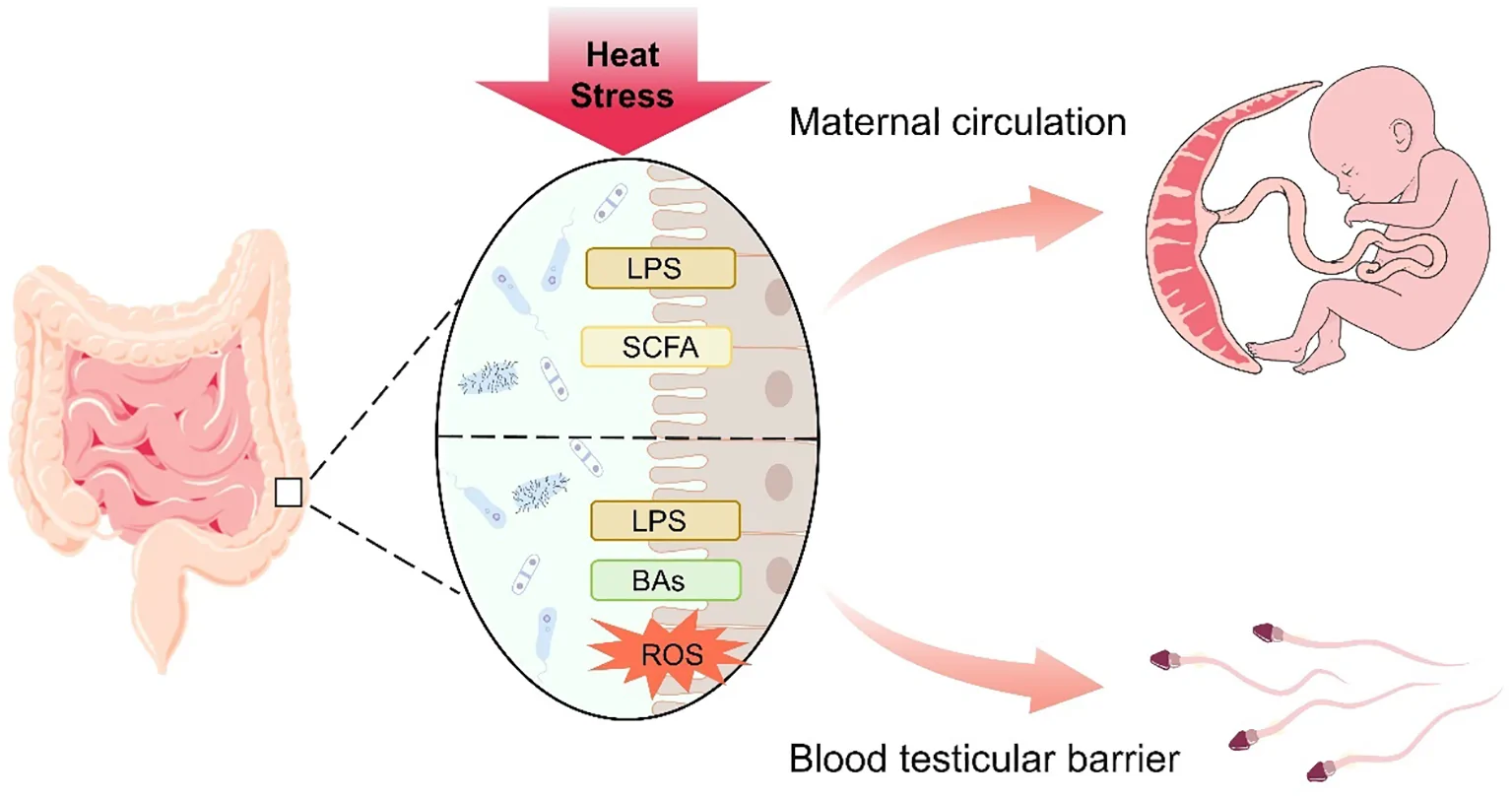
Proposed impact of GM metabolites on fetal development and spermatogenesis under HS. The diagram summarizes putative pathways through which LPS, SCFAs, BAs, and ROS may influence placental and testicular function during heat exposure. ROS, reactive oxygen species; LPS, lipopolysaccharide; SCFAs, short-chain fatty acids; BAs, bile acids.

One proposed pathway involves the translocation of gut-derived LPS into the systemic circulation (**Figure 4**). In a mouse model of systemic HS, pro-inflammatory factors and endotoxin were detected locally in the intestine and subsequently entered the blood, and probiotic intervention that reshaped the GM reduced gut inflammation and attenuated HS-induced necroptosis in germ cells (Cai et al., 2023). In humans, experimental endotoxin challenge in healthy men produced a transient inflammatory response followed by a decline in serum testosterone, providing evidence that endotoxin-driven inflammation can impair Leydig cell function. These findings suggest that HS-induced intestinal barrier disruption may facilitate LPS translocation, which in turn activates TLR4-mediated inflammatory pathways in testicular tissue and contributes to germ cell injury. However, direct evidence linking HS-induced gut dysbiosis to testicular TLR4 activation in a single experimental model remains limited (Tremellen et al., 2018; Chen et al., 2021).

Concurrently, gut dysbiosis may amplify oxidative stress in the reproductive system (**Figure 4**). Endotoxin-driven systemic inflammation recruits immune cells that generate ROS, and sperm cells are highly vulnerable to ROS-mediated injury, leading to reduced motility and viability (Du Plessis et al., 2015; Barati et al., 2020; Uchiyama et al., 2022). In male dairy goats exposed to summer HS, melatonin administration reduced inflammatory and oxidative stress levels in testicular tissue and improved sperm quality. FMT experiments confirmed that this protective effect was partially mediated by GM remodeling, with arachidonic acid-related metabolites identified as potential mediators (Reiter et al., 2002; Tan et al., 2007; Illiano et al., 2020; Guo et al., 2024). These observations support an association between gut microbial alterations and testicular oxidative injury under HS, although the causal pathway remains to be fully elucidated.

Furthermore, HS may impair male fertility through disruption of BA metabolism (**Figure 4**). In a mouse model of chronic heat exposure, HS induced GM dysbiosis characterized by altered secondary BA profiles, which in turn impaired intestinal retinol absorption. Reduced retinol availability compromised spermatogenesis, and this pathway was supported by FMT experiments demonstrating that transfer of heat-exposed microbiota reproduced the BA-retinol-spermatogenesis phenotype (He et al., 2024). This fat-soluble vitamin is essential for spermatogenesis and relies on adequate BA for its uptake (Yang et al., 2022; Fogelson et al., 2023). The resulting functional deficiency in retinol directly impacts testicular function. Consistent with this mechanism, dietary BA supplementation has been shown to modulate GM, promote retinol absorption, and enhance reproductive performance in experimental models (Zhang et al., 2022). Collectively, these findings suggest that HS may impair spermatogenesis in part through disruption of GM-associated BA and retinol metabolism, although the relative contribution of this pathway compared with direct thermal injury remains to be quantified.

#### 4.3.2 Gut-placenta axis: LPS-induced inflammation and SCFA deficiency

Pathological heat exposure sufficient to induce HS disrupts the placental barrier, increasing fetal exposure to toxins and pathogens and thereby threatening intrauterine development (Guo et al., 2021). The GM and its metabolites regulate placental function, and GM dysbiosis has been associated with adverse pregnancy outcomes (Qi et al., 2021).

One major pathway involves the translocation of gut-derived LPS (**Figure 4**). Elevated LPS levels in the maternal circulation under HS can cross the placental barrier (Gohir et al., 2019). Upon entry into the fetal compartment, LPS is recognized by TLRs expressed on placental tissues, including TLR4 on first-trimester trophoblasts (Abrahams et al., 2004; Brown et al., 2019). Binding of LPS to TLR4 initiates downstream MAPK and NF-κB signaling, promoting the synthesis of pro-inflammatory factors IL-6 and TNF-α (Miyauchi et al., 2018; Fan et al., 2019). This inflammatory response disrupts placental vascular function. Notably, active MAPK signaling has been linked to increased placental VEGF expression. Chronically elevated VEGF can cause abnormal angiogenesis, ultimately resulting in inadequate nutrient transfer between mother and fetus (Sawada et al., 2015; Rosario et al., 2021; Feng et al., 2025; Wu et al., 2025).

Concurrently, HS-induced GM dysbiosis can contribute to fetal growth restriction through a reduction in SCFAs (**Figure 4**) (Zheng et al., 2023). SCFAs exert multiple effects by signaling through G protein-coupled receptors at the maternal-placental interface, modulating host metabolism and promoting anti-inflammatory regulatory T (Treg) cell differentiation (Yang et al., 2015; Zietek et al., 2021). A decline in SCFAs during GM dysbiosis may weaken these pathways (Zheng et al., 2023). In a pregnant mouse model of chronic heat exposure, HS was associated with elevated LPS levels along the maternal intestine-placenta-fetus axis, reduced SCFA concentrations, and fetal intrauterine growth restriction (Zheng et al., 2023). FMT and pseudo-germ-free experiments in a melatonin intervention study provided causal support for a microbiota-dependent component in fetal growth restriction under high-temperature exposure (Wu et al., 2025). However, the extent to which these microbiota-dependent pathways amplify direct heat injury versus operate independently remains unclear.

### 4.4 Other organs

#### 4.4.1 Systemic inflammatory and oxidative insults in renal and cardiac injury

HS might induce acute kidney injury and myocardial injury through direct thermal cytotoxicity, splanchnic hypoperfusion, ischemia reperfusion injury, and systemic inflammatory responses (Bouchama and Knochel, 2002; Hansson and Johansson, 2010; Pearce et al., 2013; Lian et al., 2020). Intestinal barrier impairment and GM dysbiosis have been proposed as potential amplifiers of this injury cascade. A key pathogenic event is splanchnic vasoconstriction and increased intestinal permeability. This event might facilitate the translocation of bacterial endotoxins such as LPS into the systemic circulation (Bouchama et al., 1991; Lambert, 2008; Abdullah et al., 2024). This endotoxemia could trigger systemic inflammation via activation of TLR4 and NF-κB signaling. The resulting release of pro-inflammatory cytokines, including IL-6, TNF-α and IL-1β, might exacerbate renal endothelial dysfunction and cardiac inflammation (Zhang et al., 2024).

In the kidney, gut dysbiosis has been associated with the accumulation of uremic toxins (Nanto-Hara and Ohtsu, 2024). In non-heat-related kidney disease models, indoxyl sulfate, a microbial metabolite of dietary tryptophan, is taken up by renal tubular cells via organic anion transporters and activates the aryl hydrocarbon receptor pathway. This activation induces oxidative stress and inflammatory responses (Enomoto et al., 2002; Shimizu et al., 2011). This may promote expression of profibrotic genes and contribute to renal tubulointerstitial fibrosis and dysfunction (Shimizu et al., 2011; Nanto-Hara and Ohtsu, 2024). In a mouse heatstroke model, heat acclimation altered GM composition and reduced liver and kidney injury (Huang et al., 2023). However, direct evidence linking HS-induced gut dysbiosis to indoxyl sulfate accumulation and kidney injury in a single experimental model is currently limited. Most evidence for indoxyl sulfate-mediated kidney injury comes from chronic kidney disease models rather than HS models.

In the heart, studies have reported that HS is associated with alterations in GM composition. In mice exposed to 40 °C for 2 hours daily over 7 days, HS induced cardiac injury accompanied by reduced *Lactobacillus* abundance and increased *Staphylococcus*. These changes correlated with elevated oxidative stress markers, including reduced superoxide dismutase and glutathione and increased malondialdehyde (Costello et al., 2009; Schroeder and Backhed, 2016). The convergence of systemic inflammation and direct cellular toxicity contributes to diverse cell death pathways in both organs (Xue et al., 2021). In a rat model of acute HS, the traditional Chinese medicine Sheng Mai San attenuated cardiac injury, restored microbial balance, and activated the Keap1-Nrf2 pathway. Similarly, in BALB/c mice exposed to 37 °C for 7 days, *Ligilactobacillus salivarius* supplementation protected against cardiac injury and was associated with increased *Lactobacillus* and reduced *Bacteroides* abundance (Zhang et al., 2022; Yang et al., 2025). These intervention studies provide concordant evidence that microbiota modulation accompanies cardiac protection under HS, but these studies do not establish whether the protection results from direct microbial effects, anti-inflammatory actions, or both. Mechanistic studies specifically addressing gut-to-heart signaling in HS models are currently lacking.

#### 4.4.2 Peripheral metabolic dysfunction in skeletal muscle and adipose tissue

HS-induced gut dysbiosis might contribute to metabolic dysfunction in peripheral tissues through disruption of microbial metabolites. One proposed mechanism involves a reduction in butyrate, which is a SCFA produced by gut bacteria (Ke et al., 2025; Lu et al., 2025). This butyrate deficiency might impair autophagy activation, a process important for maintaining skeletal muscle homeostasis. Consequently, HS might trigger oxidative stress in skeletal muscle and disrupt muscle homeostasis through impaired autophagy and altered muscle fiber type composition (Gao et al., 2015; Ganesan et al., 2017). In a rat heatstroke model, sodium butyrate treatment attenuated multiple organ injury, including muscle damage, alongside increased *Ligilactobacillus murinus* and *Akkermansia muciniphila* and decreased *Lactobacillus johnsonii* (Ke et al., 2025). These findings suggest that butyrate deficiency might impair autophagy and contribute to skeletal muscle oxidative stress under HS, although the causal pathway from HS-induced dysbiosis to reduced butyrate production to muscle injury has not been fully established in a single longitudinal study.

In adipose tissue, gut bacteria might translocate to extra-intestinal sites under HS. In an HS-induced mouse model, HS inhibited epididymal white adipose tissue expansion and induced lipid metabolic disorder without damaging other organs such as the heart, liver, spleen, or muscle (Massier et al., 2020). Microbial profiling revealed that HS shifted the bacterial community in the cecum and epididymal white adipose tissue, with increased abundance of *Lactobacillus* in the adipose tissue. Integrated multi-omics analysis showed that the adipose tissue microbiota was associated with host lipid metabolism, and genes involved in fatty acid metabolism, including *AcadI, Cpt1b, Acaca, Fasn, and Sorbs1*, were upregulated (Patni and Garg, 2015; Chouchani and Kajimura, 2019). *Lactiplantibacillus plantarum* supplementation ameliorated HS-induced loss of epididymal white adipose tissue and dyslipidemia, and reduced gut bacterial translocation by improving gut barrier function (Wang et al., 2018; Liu et al., 2023). These observations support an association between gut microbial translocation and adipose tissue metabolic dysfunction under HS. However, FMT or gnotobiotic validation is lacking for this pathway, and the causal relationship between gut microbial translocation and adipose tissue dysfunction remains to be established.

## 5 Intervention strategies targeting the gut-organ axis to mitigate HS-induced pathology

Given the potential role of GM dysbiosis as an amplifier and mediator of the injury cascade during severe HS, the gastrointestinal tract represents a promising preventive and adjunctive therapeutic target. As outlined above, HS-induced intestinal injury involves direct thermal cytotoxicity, ischemic damage, and microbial translocation, which together create a cycle of inflammation and barrier dysfunction. Consequently, interventions that interrupt this pathogenic amplification loop by restoring microbial eubiosis, reinforcing the intestinal barrier, and counteracting systemic oxidative stress hold promise in preclinical models (Armstrong et al., 2018; Lian et al., 2020; Tang et al., 2023). Immediate cooling and supportive care remain the cornerstones of heatstroke management (Belval et al., 2018; Hosokawa et al., 2019). The strategies discussed below should be viewed as preventive or adjunctive approaches. Their evidence is derived mainly from mammalian models, with occasional supportive insights from avian studies, and their translational readiness varies considerably (Camilleri et al., 2019).

### 5.1 Direct microbiota modulation with probiotics and prebiotics

Probiotics, particularly species from the *Lactobacillus* and *Bifidobacterium* genera, have shown protective effects in several rodent and porcine models of acute and chronic HS (Reid, 2016). Their proposed mechanisms include strengthening the intestinal barrier through upregulation of tight junction proteins and enhancement of transepithelial electrical resistance, which may counteract hyperthermia-induced permeability (Ashraf et al., 2013). However, evidence from human exertional HS models is mixed. While some multistrain probiotic formulations have shown benefits in reducing endotoxemia, other single-strain interventions have failed to demonstrate significant protection (Ogden et al., 2020a; King et al., 2021). This discrepancy may be attributed to the strain-specific nature of probiotic effects and the dependence on the host’s baseline microbiome status (Jäger et al., 2019).

Prebiotics, such as galacto-oligosaccharides (GOS) and fructo-oligosaccharides (FOS), confer protection through a dual mechanism (Valcheva and Dieleman, 2016). Indirectly, they serve as fermentable substrates that selectively promote beneficial saccharolytic bacteria, thereby enhancing the production of SCFAs, which are often depleted during HS. Directly, prebiotics can interact with IECs to enhance barrier function by stimulating mucus production and upregulating junctional proteins (Jeurink et al., 2013; Bhatia et al., 2015). This combined action makes prebiotics a promising strategy for restoring both metabolic and structural homeostasis in the gut during heat exposure.

### 5.2 Barrier-supporting and cytoprotective agents

Alpha-lipoic acid (ALA) and resveratrol are two compounds with well-documented cytoprotective properties (Shila et al., 2005; Rochette et al., 2013). ALA may support the intestinal barrier during HS by maintaining junctional protein expression and preserving villus architecture. These effects have been associated with redox regulation and heat shock protein 70 (HSP70) upregulation (Lian et al., 2020). Beyond its direct antioxidant role via free-radical scavenging and Nrf2 pathway activation, ALA exhibits anti-inflammatory activity by inhibiting the NF-κB pathway (Durante, 2011; Koriyama et al., 2013). Resveratrol may also support cellular defense during HS by upregulating HSP70, heat shock protein 90 (HSP90), and heme oxygenase 1 (HO-1). Its protective effects on intestinal integrity have been demonstrated in both *in vitro* and *in vivo* models (Cao et al., 2019).

Arginine serves as a precursor for nitric oxide (NO) synthesis. NO regulates intestinal blood flow, while arginine can activate the mechanistic target of rapamycin (mTOR) pathway to promote epithelial repair and cell proliferation (Hou et al., 2020). Arginine also exerts immunomodulatory effects and has been shown to attenuate spermatogenic injury in HS mice through microbiota-dependent mechanisms supported by FMT (Wang et al., 2025).

Glutamine is a primary fuel for enterocytes and is important for preserving intestinal mucosal integrity under HS (Wu, 2010). By supporting epithelial energy metabolism and barrier repair, glutamine may indirectly stabilize host microbiota interactions, reduce luminal antigen translocation, and limit endotoxin-driven inflammatory amplification. It strengthens the barrier by upregulating tight junction and adherens junction proteins, thereby reducing paracellular permeability (Zuhl et al., 2014). A central mechanism involves the induction of heat shock proteins such as HSP70 via the activation of heat shock factor 1 (HSF-1), which enhances antioxidant capacity and stabilizes junctional complexes (Akagi et al., 2013). Furthermore, glutamine may indirectly support immune barrier function by improving epithelial metabolism, reducing permeability, maintaining tight junction integrity and limiting microbiota-derived endotoxin translocation in stress and injury models (King et al., 2021). Its efficacy in reducing exercise-induced intestinal permeability has been demonstrated in a dose-dependent manner in human studies (Ogden et al., 2020a). However, high doses may be required, and gastrointestinal tolerance can be a practical limitation (Pugh et al., 2017; Ogden et al., 2020b).

## 6 Limitations and future directions

A growing body of evidence has linked HS-associated GM dysbiosis with intestinal barrier disruption and remote organ injury. However, several limitations should be considered. First, the current evidence base is dominated by preclinical studies. These studies include acute HS, chronic HS, exertional heatstroke, and heatstroke models, which differ substantially in thermal intensity, exposure duration, hemodynamic compromise, and organ injury patterns.

Second, human evidence remains limited. Clinical heatstroke is heterogeneous and is influenced by exertional status, age, comorbidities, medications, hydration, cooling time, antibiotic exposure, nutritional support, and intensive care interventions. These factors may independently alter the GM and complicate interpretation of stool, blood, metabolomic, and inflammatory signals. Future human studies should use temporally resolved sampling during acute illness and recovery, together with microbiome sequencing, metabolomics, barrier function assays, inflammatory markers, and organ injury biomarkers.

Third, causality remains incompletely established. FMT studies provide supportive evidence for microbiota-dependent effects, but they do not identify the specific organisms, microbial products, metabolites, or host receptors responsible for tissue injury or protection. Future studies should combine gnotobiotic models, defined microbial consortia, metabolite rescue experiments, receptor blockade, and longitudinal sampling to clarify whether dysbiosis precedes, accompanies, or follows systemic injury.

Fourth, the strength of evidence differs across organ axes. The gut-liver axis is relatively well supported by anatomical, metabolic, and experimental evidence, whereas the gut-brain axis is supported by emerging but still limited mechanistic data. Evidence for the gut-reproductive, gut-kidney, gut-heart, gut-muscle, and gut-adipose axes remains more dependent on HS models and extrapolation from broader microbiome literature. These proposed pathways should therefore be interpreted according to their underlying evidence strength.

Finally, microbiome-targeted interventions remain largely preclinical. Probiotics, prebiotics, antioxidants, functional amino acids, and microbial metabolites may be more suitable as preventive or adjunctive strategies than as acute rescue therapies. Rapid cooling and supportive care remain the foundation of heatstroke management. Future studies should define the timing, dose, safety, route of administration, target population, and clinical readiness of microbiome-directed interventions.

## 7 Conclusions

**Figure 5:**
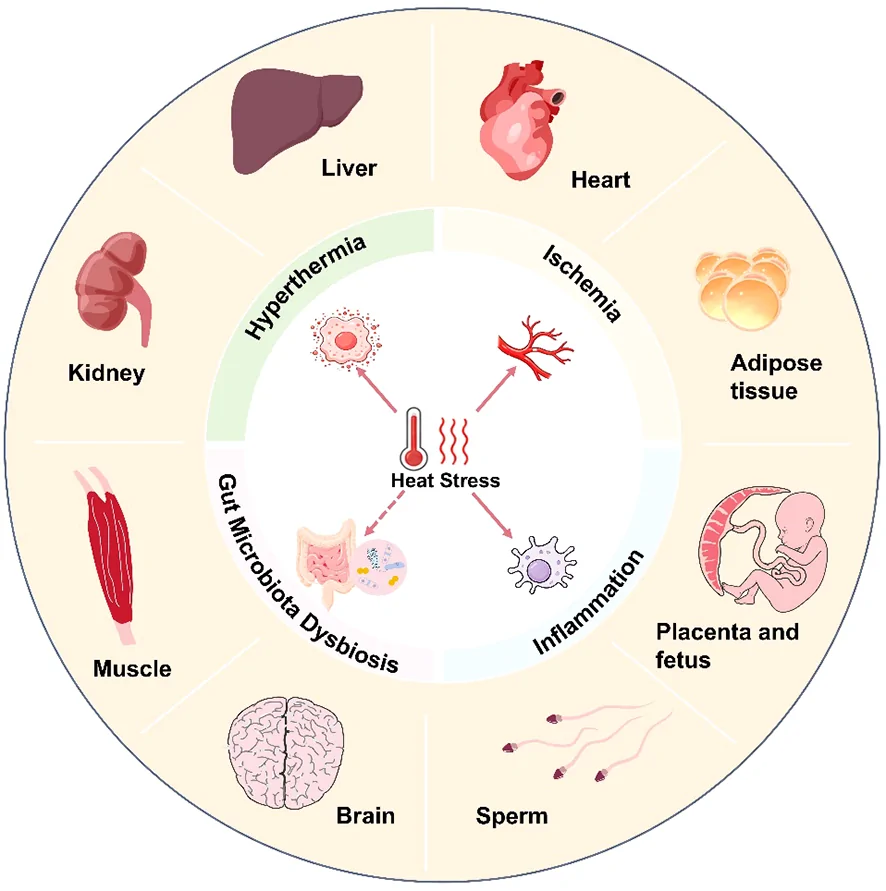
Conceptual network of heat stress-induced multi-organ injury incorporating gut microbiota dysbiosis. Heat stress is depicted as the central systemic insult, with hyperthermia, ischemia, inflammation, and gut microbiota dysbiosis representing interconnected components of the injury network. These processes may contribute to pathological changes in the liver, heart, adipose tissue, placenta and fetus, male reproductive system, brain, skeletal muscle, and kidney. The circular organization highlights the systemic nature of heat injury and positions gut microbiota dysbiosis as a potential mediator and amplifier within a broader multifactorial network. Arrows indicate the proposed relationships between heat stress and the four central components, whereas the outer ring denotes the organs and tissues potentially affected.

This review supports the concept that HS-induced GM dysbiosis may act as a mediator and amplifier of multi-organ injury within a broader heat injury network, linking specific microbial communities and their metabolic outputs to distant organ pathology (**Figure 5**). However, the strength of evidence for this framework is not uniform. The gut-liver and gut-brain axes are supported by robust evidence, including FMT and gnotobiotic studies establishing microbiota-dependent amplification of intestinal barrier failure, hepatic inflammation, and neuroinflammation. In contrast, evidence for the gut-reproductive, gut-kidney, gut-heart, gut-muscle, and gut-adipose axes remains predominantly associative or derived from interventional correlations without formal causality testing. Key mechanisms implicated in these pathways extend beyond the classical LPS-TLR4 axis to encompass short-chain fatty acid deficiency, BA dysregulation, and oxidative stress.

Causality remains incompletely established for many proposed pathways; FMT studies support microbiota-dependent effects but do not consistently identify the specific organisms, metabolites, or host receptors responsible for tissue injury or protection. Consequently, therapeutic strategies targeting the gut microbiome through probiotics, prebiotics, or specific metabolites represent a promising but largely preclinical frontier. These strategies should be viewed as adjunctive to cooling and supportive care, which remain the foundation of heatstroke management. Future research should prioritize temporally resolved human studies, multi-omics integration, and causal validation in defined experimental models to support translation of these mechanistic insights into targeted interventions.
