# Supplementary material for: Heat stress-induced gut dysbiosis and multi-organ injury: mechanisms and therapeutic modulation
Source: Front Cell Infect Microbiol. 2026 Sep 9;16:1930660. doi: 10.3389/fcimb.2026.1930660 (PMC13598704; doi:10.3389/fcimb.2026.1930660)
Supplement: Supplementary file 1 [file Supplementaryfile1.docx]

**Supplementary File S1: Search Strategy (From inception to June 2026)**

|  | **Organ** | **Database** | **Search Strategy** | **No. of Articles** |
| --- | --- | --- | --- | --- |
|  | **Liver** | Pubmed | ("Heat Stress"[Mesh] OR "Heat-Shock Response"[Mesh] OR heat stress*[tiab] OR heatstroke[tiab] OR heat stroke[tiab] OR hyperthermia[tiab]) AND ("Gastrointestinal Microbiome"[Mesh] OR "Microbiota"[Mesh] OR gut microbiota[tiab] OR gut microbiome[tiab] OR intestinal microbiota[tiab] OR intestinal flora[tiab] OR dysbiosis[tiab]) AND ("Liver"[Mesh] OR "Liver Diseases"[Mesh] OR liver[tiab] OR hepatic[tiab] OR hepatocyte*[tiab]) | 49 |
|  |  | Web of Science | TS=("heat stress" OR "heat-shock response" OR heatstroke OR "heat stroke" OR hyperthermia) AND TS=("gut microbiota" OR "gut microbiome" OR "intestinal microbiota" OR "intestinal flora" OR dysbiosis OR microbiome) AND TS=(liver OR hepatic OR hepatocyte*) | 96 |
|  |  | Scopus | TITLE-ABS-KEY(( "heat stress" OR "heat-shock response" OR heatstroke OR "heat stroke" OR hyperthermia ) AND ( "gut microbiota" OR "gut microbiome" OR "intestinal microbiota" OR "intestinal flora" OR dysbiosis OR microbiome ) AND ( liver OR hepatic OR hepatocyte* )) | 88 |
|  |  | Embase | ('heat stress'/exp OR 'heat shock response'/exp OR 'heatstroke'/exp OR 'hyperthermia'/exp OR 'heat stress':ti,ab,kw OR 'heatstroke':ti,ab,kw OR 'heat stroke':ti,ab,kw OR 'hyperthermia':ti,ab,kw) AND ('gut microbiome'/exp OR 'microbiota'/exp OR 'intestinal flora'/exp OR 'dysbiosis'/exp OR 'gut microbiota':ti,ab,kw OR 'gut microbiome':ti,ab,kw OR 'intestinal microbiota':ti,ab,kw OR 'intestinal flora':ti,ab,kw OR 'dysbiosis':ti,ab,kw) AND ('liver'/exp OR 'liver disease'/exp OR liver:ti,ab,kw OR hepatic:ti,ab,kw OR hepatocyte*:ti,ab,kw) | 83 |
|  | **Brain** | Pubmed | ("Heat Stress"[Mesh] OR "Heat-Shock Response"[Mesh] OR heat stress*[tiab] OR heatstroke[tiab] OR heat stroke[tiab] OR hyperthermia[tiab]) AND ("Gastrointestinal Microbiome"[Mesh] OR "Microbiota"[Mesh] OR gut microbiota[tiab] OR gut microbiome[tiab] OR intestinal microbiota[tiab] OR intestinal flora[tiab] OR dysbiosis[tiab]) AND ("Brain"[Mesh] OR "Brain Diseases"[Mesh] OR brain[tiab] OR cerebral[tiab] OR encephalon[tiab] OR neuroinflammation[tiab] OR "Blood-Brain Barrier"[Mesh] OR blood-brain barrier[tiab] OR BBB[tiab]) | 21 |
|  |  | Web of Science | TS=("heat stress" OR "heat-shock response" OR heatstroke OR "heat stroke" OR hyperthermia) AND TS=("gut microbiota" OR "gut microbiome" OR "intestinal microbiota" OR "intestinal flora" OR dysbiosis OR microbiome) AND TS=(brain OR cerebral OR encephalon OR neuroinflammation OR "blood-brain barrier" OR BBB) | 51 |
|  |  | Scopus | TITLE-ABS-KEY(( "heat stress" OR "heat-shock response" OR heatstroke OR "heat stroke" OR hyperthermia ) AND ( "gut microbiota" OR "gut microbiome" OR "intestinal microbiota" OR "intestinal flora" OR dysbiosis OR microbiome ) AND ( brain OR cerebral OR encephalon OR neuroinflammation OR "blood-brain barrier" OR BBB )) | 46 |
|  |  | Embase | ('heat stress'/exp OR 'heat shock response'/exp OR 'heatstroke'/exp OR 'hyperthermia'/exp OR 'heat stress':ti,ab,kw OR 'heatstroke':ti,ab,kw OR 'heat stroke':ti,ab,kw OR 'hyperthermia':ti,ab,kw) AND ('gut microbiome'/exp OR 'microbiota'/exp OR 'intestinal flora'/exp OR 'dysbiosis'/exp OR 'gut microbiota':ti,ab,kw OR 'gut microbiome':ti,ab,kw OR 'intestinal microbiota':ti,ab,kw OR 'intestinal flora':ti,ab,kw OR 'dysbiosis':ti,ab,kw) AND ('brain'/exp OR 'brain disease'/exp OR brain:ti,ab,kw OR cerebral:ti,ab,kw OR encephalon:ti,ab,kw OR neuroinflammation:ti,ab,kw OR 'blood-brain barrier':ti,ab,kw OR bbb:ti,ab,kw) | 109 |
|  | **Reproductive System** | Pubmed | ("Heat Stress"[Mesh] OR "Heat-Shock Response"[Mesh] OR heat stress*[tiab] OR heatstroke[tiab] OR heat stroke[tiab] OR hyperthermia[tiab]) AND ("Gastrointestinal Microbiome"[Mesh] OR "Microbiota"[Mesh] OR gut microbiota[tiab] OR gut microbiome[tiab] OR intestinal microbiota[tiab] OR intestinal flora[tiab] OR dysbiosis[tiab]) AND ("Testis"[Mesh] OR "Spermatozoa"[Mesh] OR "Uterus"[Mesh] OR "Placenta"[Mesh] OR "Fetus"[Mesh] OR testis[tiab] OR testes[tiab] OR testicular[tiab] OR seminiferous[tiab] OR sperm[tiab] OR spermatozoa[tiab] OR spermatogenesis[tiab] OR "sperm quality"[tiab] OR "sperm motility"[tiab] OR uterus[tiab] OR uterine[tiab] OR endometrium[tiab] OR myometrium[tiab] OR placenta[tiab] OR placental[tiab] OR trophoblast[tiab] OR "placental insufficiency"[tiab] OR fetus[tiab] OR fetal[tiab] OR foetus[tiab] OR foetal[tiab] OR intrauterine[tiab] OR "fetal growth"[tiab] OR "Reproduction"[Mesh] OR reproduction[tiab] OR reproductive[tiab] OR "male reproductive system"[tiab] OR "female reproductive system"[tiab] OR gonad[tiab] OR gonadal[tiab]) | 33 |
|  |  | Web of Science | TS=("heat stress" OR "heat-shock response" OR heatstroke OR "heat stroke" OR hyperthermia) AND TS=("gut microbiota" OR "gut microbiome" OR "intestinal microbiota" OR "intestinal flora" OR dysbiosis OR microbiome) AND TS=(testis OR testes OR testicular OR seminiferous OR sperm OR spermatozoa OR spermatogenesis OR "sperm quality" OR "sperm motility" OR uterus OR uterine OR endometrium OR myometrium OR placenta OR placental OR trophoblast OR "placental insufficiency" OR fetus OR fetal OR foetus OR foetal OR intrauterine OR "fetal growth" OR reproduction OR reproductive OR gonad OR gonadal) | 135 |
|  |  | Scopus | TITLE-ABS-KEY(( "heat stress" OR "heat-shock response" OR heatstroke OR "heat stroke" OR hyperthermia ) AND ( "gut microbiota" OR "gut microbiome" OR "intestinal microbiota" OR "intestinal flora" OR dysbiosis OR microbiome ) AND ( testis OR testes OR testicular OR seminiferous OR sperm OR spermatozoa OR spermatogenesis OR "sperm quality" OR "sperm motility" OR uterus OR uterine OR endometrium OR myometrium OR placenta OR placental OR trophoblast OR "placental insufficiency" OR fetus OR fetal OR foetus OR foetal OR intrauterine OR "fetal growth" OR reproduction OR reproductive OR gonad OR gonadal )) | 53 |
|  |  | Embase | ('heat stress'/exp OR 'heat shock response'/exp OR 'heatstroke'/exp OR 'hyperthermia'/exp OR 'heat stress':ti,ab,kw OR 'heatstroke':ti,ab,kw OR 'heat stroke':ti,ab,kw OR 'hyperthermia':ti,ab,kw) AND ('gut microbiome'/exp OR 'microbiota'/exp OR 'intestinal flora'/exp OR 'dysbiosis'/exp OR 'gut microbiota':ti,ab,kw OR 'gut microbiome':ti,ab,kw OR 'intestinal microbiota':ti,ab,kw OR 'intestinal flora':ti,ab,kw OR 'dysbiosis':ti,ab,kw) AND ('testis'/exp OR 'spermatozoon'/exp OR 'uterus'/exp OR 'placenta'/exp OR 'fetus'/exp OR testis:ti,ab,kw OR testes:ti,ab,kw OR testicular:ti,ab,kw OR seminiferous:ti,ab,kw OR sperm:ti,ab,kw OR spermatozoa:ti,ab,kw OR spermatogenesis:ti,ab,kw OR 'sperm quality':ti,ab,kw OR 'sperm motility':ti,ab,kw OR uterus:ti,ab,kw OR uterine:ti,ab,kw OR endometrium:ti,ab,kw OR myometrium:ti,ab,kw OR placenta:ti,ab,kw OR placental:ti,ab,kw OR trophoblast:ti,ab,kw OR 'placental insufficiency':ti,ab,kw OR fetus:ti,ab,kw OR fetal:ti,ab,kw OR foetus:ti,ab,kw OR foetal:ti,ab,kw OR intrauterine:ti,ab,kw OR 'fetal growth':ti,ab,kw) | 31 |
|  | **Muscle** | Pubmed | ("Heat Stress"[Mesh] OR "Heat-Shock Response"[Mesh] OR heat stress*[tiab] OR heatstroke[tiab] OR heat stroke[tiab] OR hyperthermia[tiab]) AND ("Gastrointestinal Microbiome"[Mesh] OR "Microbiota"[Mesh] OR gut microbiota[tiab] OR gut microbiome[tiab] OR intestinal microbiota[tiab] OR intestinal flora[tiab] OR dysbiosis[tiab]) AND ("Muscles"[Mesh] OR "Muscular Diseases"[Mesh] OR muscle[tiab] OR muscles[tiab] OR muscular[tiab] OR skeletal muscle[tiab] OR myocyte[tiab] OR "Muscle, Skeletal"[Mesh]) | 12 |
|  |  | Web of Science | TS=("heat stress" OR "heat-shock response" OR heatstroke OR "heat stroke" OR hyperthermia) AND TS=("gut microbiota" OR "gut microbiome" OR "intestinal microbiota" OR "intestinal flora" OR dysbiosis OR microbiome) AND TS=(muscle OR muscles OR muscular OR "skeletal muscle" OR myocyte) | 65 |
|  |  | Scopus | TITLE-ABS-KEY ( ( "heat stress" OR "heat-shock response" OR heatstroke OR "heat stroke" OR hyperthermia ) AND ( "gut microbiota" OR "gut microbiome" OR "intestinal microbiota" OR "intestinal flora" OR dysbiosis OR microbiome ) AND ( muscle OR muscles OR muscular OR "skeletal muscle" OR myocyte ) ) | 36 |
|  |  | Embase | ('heat stress'/exp OR 'heat shock response'/exp OR 'heatstroke'/exp OR 'hyperthermia'/exp OR 'heat stress':ti,ab,kw OR 'heatstroke':ti,ab,kw OR 'heat stroke':ti,ab,kw OR 'hyperthermia':ti,ab,kw) AND ('gut microbiome'/exp OR 'microbiota'/exp OR 'intestinal flora'/exp OR 'dysbiosis'/exp OR 'gut microbiota':ti,ab,kw OR 'gut microbiome':ti,ab,kw OR 'intestinal microbiota':ti,ab,kw OR 'intestinal flora':ti,ab,kw OR 'dysbiosis':ti,ab,kw) AND ('muscle'/exp OR 'muscle disease'/exp OR 'skeletal muscle'/exp OR muscle:ti,ab,kw OR muscles:ti,ab,kw OR muscular:ti,ab,kw OR 'skeletal muscle':ti,ab,kw OR myocyte:ti,ab,kw) | 39 |
|  | **Adipose Tissue** | Pubmed | ("Heat Stress"[Mesh] OR "Heat-Shock Response"[Mesh] OR heat stress*[tiab] OR heatstroke[tiab] OR heat stroke[tiab] OR hyperthermia[tiab]) AND ("Gastrointestinal Microbiome"[Mesh] OR "Microbiota"[Mesh] OR gut microbiota[tiab] OR gut microbiome[tiab] OR intestinal microbiota[tiab] OR intestinal flora[tiab] OR dysbiosis[tiab]) AND ("Adipose Tissue"[Mesh] OR adipose[tiab] OR adipocyte[tiab] OR adipogenesis[tiab] OR "Adipocytes"[Mesh] OR fat[tiab] OR "White Adipose Tissue"[Mesh] OR "Brown Adipose Tissue"[Mesh]) | 30 |
|  |  | Web of Science | TS=("heat stress" OR "heat-shock response" OR heatstroke OR "heat stroke" OR hyperthermia) AND TS=("gut microbiota" OR "gut microbiome" OR "intestinal microbiota" OR "intestinal flora" OR dysbiosis OR microbiome) AND TS=(adipose OR adipocyte OR adipogenesis OR fat OR "white adipose" OR "brown adipose") | 91 |
|  |  | Scopus | TITLE-ABS-KEY(( "heat stress" OR "heat-shock response" OR heatstroke OR "heat stroke" OR hyperthermia ) AND ( "gut microbiota" OR "gut microbiome" OR "intestinal microbiota" OR "intestinal flora" OR dysbiosis OR microbiome ) AND ( adipose OR adipocyte OR adipogenesis OR fat OR "white adipose" OR "brown adipose" )) | 64 |
|  |  | Embase | ('heat stress'/exp OR 'heat shock response'/exp OR 'heatstroke'/exp OR 'hyperthermia'/exp OR 'heat stress':ti,ab,kw OR 'heatstroke':ti,ab,kw OR 'heat stroke':ti,ab,kw OR 'hyperthermia':ti,ab,kw) AND ('gut microbiome'/exp OR 'microbiota'/exp OR 'intestinal flora'/exp OR 'dysbiosis'/exp OR 'gut microbiota':ti,ab,kw OR 'gut microbiome':ti,ab,kw OR 'intestinal microbiota':ti,ab,kw OR 'intestinal flora':ti,ab,kw OR 'dysbiosis':ti,ab,kw) AND ('adipose tissue'/exp OR adipose:ti,ab,kw OR adipocyte:ti,ab,kw OR adipogenesis:ti,ab,kw OR fat:ti,ab,kw OR 'white adipose':ti,ab,kw OR 'brown adipose':ti,ab,kw) | 61 |
|  | **Heart** | Pubmed | ("Heat Stress"[Mesh] OR "Heat-Shock Response"[Mesh] OR heat stress*[tiab] OR heatstroke[tiab] OR "heat stroke"[tiab] OR hyperthermia[tiab]) AND ("Gastrointestinal Microbiome"[Mesh] OR "Microbiota"[Mesh] OR "gut microbiota"[tiab] OR "gut microbiome"[tiab] OR "intestinal microbiota"[tiab] OR "intestinal flora"[tiab] OR dysbiosis[tiab]) AND ("Kidney"[Mesh] OR "Kidney Diseases"[Mesh] OR "Renal Insufficiency"[Mesh] OR kidney[tiab] OR renal[tiab] OR nephron[tiab] OR glomerular[tiab] OR glomerulus[tiab] OR nephro[tiab]) | 15 |
|  |  | Web of Science | TS=("heat stress" OR "heat-shock response" OR heatstroke OR "heat stroke" OR hyperthermia) AND TS=("gut microbiota" OR "gut microbiome" OR "intestinal microbiota" OR "intestinal flora" OR dysbiosis OR microbiome) AND TS=(kidney OR renal OR nephron OR glomerular OR glomerulus OR nephro) | 39 |
|  |  | Scopus | TITLE-ABS-KEY(( "heat stress" OR "heat-shock response" OR heatstroke OR "heat stroke" OR hyperthermia ) AND ( "gut microbiota" OR "gut microbiome" OR "intestinal microbiota" OR "intestinal flora" OR dysbiosis OR microbiome ) AND ( heart OR cardiac OR myocardial OR myocardium OR cardiomyocyte OR cardiovascular OR cardio OR coronary )) | 38 |
|  |  | Embase | ('heat stress'/exp OR 'heat shock response'/exp OR 'heatstroke'/exp OR 'hyperthermia'/exp OR 'heat stress':ti,ab,kw OR 'heatstroke':ti,ab,kw OR 'heat stroke':ti,ab,kw OR 'hyperthermia':ti,ab,kw) AND ('gut microbiome'/exp OR 'microbiota'/exp OR 'intestinal flora'/exp OR 'dysbiosis'/exp OR 'gut microbiota':ti,ab,kw OR 'gut microbiome':ti,ab,kw OR 'intestinal microbiota':ti,ab,kw OR 'intestinal flora':ti,ab,kw OR 'dysbiosis':ti,ab,kw) AND ('heart'/exp OR 'heart disease'/exp OR 'cardiovascular disease'/exp OR heart:ti,ab,kw OR cardiac:ti,ab,kw OR myocardial:ti,ab,kw OR myocardium:ti,ab,kw OR cardiomyocyte:ti,ab,kw OR cardiovascular:ti,ab,kw OR cardio:ti,ab,kw OR coronary:ti,ab,kw) | 81 |
| **Kidney** | | Pubmed | ("Heat Stress"[Mesh] OR "Heat-Shock Response"[Mesh] OR heat stress[tiab] OR heatstroke[tiab] OR "heat stroke"[tiab] OR hyperthermia[tiab]) AND ("Gastrointestinal Microbiome"[Mesh] OR "Microbiota"[Mesh] OR "gut microbiota"[tiab] OR "gut microbiome"[tiab] OR "intestinal microbiota"[tiab] OR "intestinal flora"[tiab] OR dysbiosis[tiab]) AND ("Kidney"[Mesh] OR "Kidney Diseases"[Mesh] OR "Acute Kidney Injury"[Mesh] OR "Renal Insufficiency"[Mesh] OR kidney[tiab] OR renal[tiab] OR nephro[tiab] OR glomerular[tiab] OR tubular[tiab] OR nephron[tiab]) | 9 |
|  |  | Web of Science | TS=("heat stress" OR "heat-shock response" OR heatstroke OR "heat stroke" OR hyperthermia) AND TS=("gut microbiota" OR "gut microbiome" OR "intestinal microbiota" OR "intestinal flora" OR dysbiosis OR microbiome) AND TS=(kidney OR renal OR nephro* OR glomerular OR tubular OR nephron OR "acute kidney injury" OR "renal insufficiency") | 25 |
|  |  | Scopus | TITLE-ABS-KEY(( "heat stress" OR "heat-shock response" OR heatstroke OR "heat stroke" OR hyperthermia ) AND ( "gut microbiota" OR "gut microbiome" OR "intestinal microbiota" OR "intestinal flora" OR dysbiosis OR microbiome ) AND ( kidney OR renal OR nephro* OR glomerular OR tubular OR nephron OR "acute kidney injury" OR "renal insufficiency" )) | 28 |
|  |  | Embase | ('heat stress'/exp OR 'heat shock response'/exp OR 'heatstroke'/exp OR 'hyperthermia'/exp OR 'heat stress':ti,ab,kw OR 'heatstroke':ti,ab,kw OR 'heat stroke':ti,ab,kw OR 'hyperthermia':ti,ab,kw) AND ('gut microbiome'/exp OR 'microbiota'/exp OR 'intestinal flora'/exp OR 'dysbiosis'/exp OR 'gut microbiota':ti,ab,kw OR 'gut microbiome':ti,ab,kw OR 'intestinal microbiota':ti,ab,kw OR 'intestinal flora':ti,ab,kw OR 'dysbiosis':ti,ab,kw) AND ('kidney'/exp OR 'kidney disease'/exp OR 'acute kidney injury'/exp OR 'renal insufficiency'/exp OR kidney:ti,ab,kw OR renal:ti,ab,kw OR nephro*:ti,ab,kw OR glomerular:ti,ab,kw OR tubular:ti,ab,kw OR nephron:ti,ab,kw) | 63 |
